# Complete Genome Sequences of *Azotobacter vinelandii* Wild-Type Strain CA and Tungsten-Tolerant Mutant Strain CA6

**DOI:** 10.1128/genomeA.00313-13

**Published:** 2013-06-06

**Authors:** Jesse D. Noar, José M. Bruno-Bárcena

**Affiliations:** North Carolina State University, Raleigh, North Carolina, USA

## Abstract

We report the complete genome sequences of *Azotobacter vinelandii* mutant strain CA6 and its parent wild-type strain, CA. When fixing nitrogen, strain CA6 displays slow growth and impaired molybdate uptake, tolerance to tungstates, and production of hydrogen gas, compared to results for strain CA. Comparing these genome sequences may provide a genetic basis for these mutant phenotypes.

## GENOME ANNOUNCEMENT

*Azotobacter vinelandii* is a Gram-negative, soil-dwelling, obligately aerobic diazotroph that was discovered more than a century ago (1). *A. vinelandii* strain CA (or OP) (ATCC 13705) was isolated as a nongummy pigment-producing wild-type strain that is easier to study than “gummy” polysaccharide-producing strains (2). A consortium of researchers has published the complete genome sequence of a variant of strain CA, a high-frequency transforming strain called DJ (ATCC BAA-1303) (3).

*A. vinelandii* strain CA6 is a spontaneous mutant strain derived from strain CA. Tungstate prevents nitrogen fixation and growth of the strain CA, but it does not inhibit CA6 (4). Strain CA6 also displays impaired molybdate uptake (5) and has been found to produce large quantities of hydrogen gas when fixing nitrogen (P. Bishop, T. Loveless, J. Olson, and J. M. Bruno-Bárcena, unpublished data). For this reason, we sequenced the genomes of both CA6 and its parent, CA, to identify the genetic bases of these mutant phenotypes.

The Microbiome Core Facility at the University of North Carolina—Chapel Hill generated shotgun sequence data from each strain using a combination of 454 GS FLX Titanium+ and Ion Torrent PGM techniques, performed according to the manufacturers’ instructions. These generated 1,070,910 reads for CA, averaging 416 bp per read, and 1,020,357 reads for CA6, averaging 414 bp per read. Reads were assembled with Geneious (Biomatters, New Zealand) using the genome sequence of strain DJ as a reference (accession no. NC_012560.1). PCR followed by dye terminator sequencing (Eton BioScience, Research Triangle Park, NC) was used to close gaps and confirm or reject uncertain variations identified *in silico*. Annotations were copied from the GenBank record of the genome of strain DJ and were modified as necessary.

### Nucleotide sequence accession numbers. 

The genome sequences of *A. vinelandii* strains CA and CA6 have been deposited in DDBJ/EMBL/GenBank under the accession no. CP005094 (CA) and CP005095 (CA6). The versions described in this paper are the first versions, accession no. CP005094 (CA) and CP005095 (CA6).
